# Revisiting RAS family GTPase signaling: effector selectivity and oncogenic bypass

**DOI:** 10.1042/BCJ20250142

**Published:** 2026-04-22

**Authors:** Dhirendra K. Simanshu, Frank McCormick

**Affiliations:** 1 NCI RAS Initiative, Cancer Research Technology Program, Frederick National Laboratory for Cancer Research, Frederick, MD, U.S.A.; 2 Helen Diller Family Comprehensive Cancer Center, University of California, San Francisco, San Francisco, CA, U.S.A.

**Keywords:** KRAS, phosphoinositide 3-kinase, RAF1, Rap1, RAS, RRAS2

## Abstract

Distinct effector-binding preferences among RAS family GTPases challenge the longstanding view that canonical RAS proteins uniformly bind and activate RAF, PI3Kα, RalGDS, and other downstream effectors. Quantitative binding data, supported by structural insights into effector recognition, instead reveal a division of labor: the canonical RAS subfamily (KRAS, HRAS, NRAS) binds RAF kinases with high affinity, the RRAS subfamily (RRAS2 and MRAS) preferentially engages PI3Kα, and the RAP subfamily (RAP1A and RAP1B) shows the strongest binding to RalGDS. These intrinsic preferences, encoded in the switch regions and further shaped by isoform and effector expression, as well as subcellular localization, establish a hierarchy in which canonical RAS, RRAS2/MRAS, and RAP1A/B primarily activate RAF, PI3Kα, and RalGDS, respectively, in normal cells. Oncogenic mutations at codons G12, G13, or Q61 disrupt this hierarchy by driving sustained accumulation of GTP-bound canonical RAS, enabling engagement of lower-affinity effectors such as PI3Kα and RalGDS. In addition, certain mutations, including KRAS-G12D and -G12V, modestly enhance PI3Kα binding, representing a neomorphic expansion of effector engagement. Together, these effects bypass intrinsic effector selectivity, allowing canonical RAS to co-opt effectors normally associated with other RAS subfamilies and broaden downstream signaling. This framework explains how inherent effector preferences govern normal signaling and how oncogenic mutations override these constraints to expand effector engagement in RAS-driven cancers.

## Introduction

**Figure 1:**
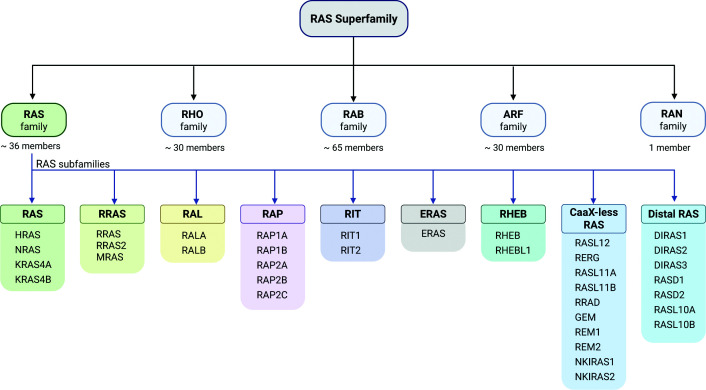
Hierarchical classification of the human RAS superfamily, families, and subfamilies The human RAS superfamily comprises over 160 small GTPases, organized into five major families based on sequence homology and function: RAS, RHO, RAB, ARF, and RAN. The RAS family, which controls gene expression, cell proliferation, and survival, is further divided into the RAS, RRAS, RAL, RAP, RIT, ERAS, and RHEB subfamilies. A subset of 17 poorly characterized RAS family GTPases, often downregulated in cancer, is further classified into distal RAS and CaaX-less RAS subfamilies, the latter lacking the canonical C-terminal prenylation motif.

The canonical RAS proteins HRAS, NRAS, and KRAS, alternatively spliced as KRAS4A and KRAS4B, act as molecular switches that translate extracellular signals into specific intracellular signaling cascades by engaging defined downstream effectors. Through these pathways, they regulate processes critical for proliferation, survival, differentiation, and migration in both normal physiology and cancer. These proteins belong to the RAS superfamily, which comprises more than 160 small GTPases, organized into the RAS, RHO, RAB, ARF, and RAN families based on sequence homology and functional specialization [1–3] (Figure 1). Collectively, these GTPases control diverse cellular processes, including signal transduction, cytoskeletal regulation, vesicular trafficking, and nucleocytoplasmic transport, highlighting their central role in cellular homeostasis. Despite this functional diversity, all family members share a conserved GTP-binding domain and operate through a common switching mechanism that cycles between an inactive GDP-bound state and an active GTP-bound state [4]. This cycling is driven by guanine nucleotide exchange factors (GEFs), which promote GDP release and GTP loading, and is terminated by GTPase-activating proteins (GAPs), which accelerate GTP hydrolysis [5]. This conserved mechanism supports a wide range of cellular functions while enabling family- and isoform-specific functions.

The human RAS family comprises 36 small GTPases organized into multiple subfamilies, with high sequence conservation within subfamilies and ∼30%–60% identity across more distantly related members [1–3] (Figure 1). The canonical RAS subfamily comprises the proto-oncogenes KRAS, HRAS, and NRAS, which signal primarily through the RAF–MAPK pathway to drive phosphorylation of numerous downstream substrates and regulate proliferation, differentiation, migration, and other essential biological processes [6]. Additional subfamilies include the RRAS subfamily, which consists of RRAS, RRAS2 (TC21), and MRAS (RRAS3); the RAP subfamily, comprising RAP1A/B and RAP2A/B/C; the RIT subfamily, containing RIT1 and RIT2; the RAL subfamily, including RALA and RALB; the ERAS subfamily, represented by ERAS; and the RHEB subfamily, consisting of RHEB and RHEBL1 [7–12] (Figure 1). These subfamilies differ in sequence features and effector-binding preferences, and many exhibit differences in subcellular localization, together contributing to isoform-specific signaling outputs. Among these, RAP proteins are of particular interest as one of them, RAP1A, was discovered in a screen for tumor suppressors [13]. The protein, initially named K-Rev1, is thought to compete with KRAS for binding to RAF and other effectors, forming non-productive complexes [14]. Tumor-suppressive effects have also been reported for wild-type HRAS, which can inhibit transformation driven by oncogenic HRAS, likely through interference with effector interactions [15]. Similarly, wild-type KRAS has been shown to suppress oncogenic KRAS-driven transformation [16], although the underlying mechanisms remain poorly defined.

Additional poorly characterized RAS proteins further diversify the family’s functional repertoire, including 17 RAS family GTPases, often down-regulated in cancer, that cluster into distal RAS and CaaX-less RAS subfamilies and may have a tumor-suppressive role [17]. For example, DIRAS3 is silenced in ovarian and breast cancers [18] and binds oncogenic RAS to disrupt nanoclustering and dampen MAPK/PI3K signaling, suppressing proliferation and promoting autophagy [19–21]. Similarly, NKIRAS1/2 limit tumorigenesis by reducing RAL-GTP and inhibiting NF-κB signaling [22]. Together, these findings indicate that distal and CaaX-less RAS GTPases act as negative modulators of canonical RAS-driven pathways.

## RAS–effector signaling

Following the discovery of HRAS as an oncogene in human tumors in 1982, the molecular mechanisms underlying RAS signaling remained largely unresolved for more than a decade [23]. A conceptual breakthrough came in 1993, when multiple groups independently identified RAF kinases as the first direct RAS effectors, showing that GTP-bound RAS recruits RAF to the plasma membrane to activate the MAPK cascade [24–28]. Shortly thereafter, PI3K [29] and RalGDS [30] were identified as additional distinct potential effectors. Subsequently, many studies have expanded the potential RAS effector repertoire to include RGL1–4, Tiam1, PLCε, AF6/AFDN/Afadin, RIN1, NORE1/RASSF family proteins, GRB14, SIN1, and hexokinase 1, among others [31].

**Figure 2:**
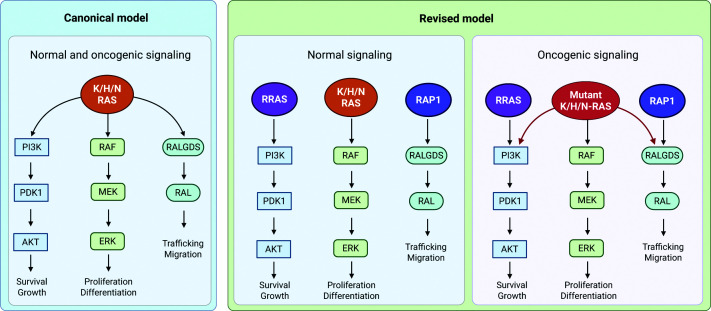
RAS signaling models in normal and oncogenic contexts Traditionally, KRAS, HRAS, and NRAS have been viewed as the primary RAS GTPases mediating RAF, PI3K, and RALGDS signaling in both normal physiology and cancer. Emerging experimental data increasingly show a more specialized division of labor: in normal cells, RAS subfamily GTPases (KRAS, HRAS, NRAS) predominantly activate RAF–MEK–ERK; RRAS/ERAS subfamily GTPases (RRAS2, MRAS, ERAS) mainly stimulate PI3Kα–AKT; and RAP subfamily GTPases (RAP1A, RAP1B) regulate RALGDS–RAL. In oncogenic contexts, RAS mutations impair intrinsic and GAP-mediated GTP hydrolysis, causing a substantial increase in GTP-bound RAS levels and enabling engagement of lower-affinity effectors such as PI3Kα and RALGDS, thereby activating the PI3K–AKT and RALGDS–RAL pathways (maroon arrows), while RRAS and RAP1 subfamily GTPases continue to regulate these pathways. This aberrant multi-effector signaling drives enhanced survival, proliferation, and migration in tumor cells.

Historically, the three canonical RAS subfamily proteins KRAS, HRAS, and NRAS were considered the primary drivers of RAS signaling, with their GTP-bound forms assumed to uniformly activate all major downstream effector pathways at the plasma membrane [32,33]. In this canonical, classical model, KRAS, HRAS, and NRAS have long been seen as broadly engaging multiple effectors: RAF kinases in MAPK signaling, PI3Kα in the PI3K–AKT pathway, and RALGDS/RGL family members in RALA/B signaling (Figure 2). This assumption that canonical RAS isoforms could simultaneously engage multiple effectors shaped much of the early research on RAS biology, influencing mechanistic studies, the design of experimental model systems, and the initial strategies for targeting RAS-driven cancers therapeutically. Early observations that RAS family members interact with different effectors with varying affinities raised questions about the specificity and physiological relevance of these interactions, highlighting the need for quantitative analysis and careful consideration of subcellular localization [34]. Over time, experimental evidence has challenged the notion of uniform effector engagement, revealing that RAS–effector interactions are more selective and context-dependent than previously appreciated, highlighting RAS signaling as a network of preferential interactions rather than uniform interactions [35]. Furthermore, almost all early studies on effectors used RAS oncogenic mutants, such as G12V or Q61L/R. This was partly due to the importance of these variants in cancer biology and partly because their stable association with GTP enabled biochemical analysis of effector complexes, unlike the transient associations observed with their wild-type counterparts. These studies identified effectors that RAS proteins could potentially engage but could not specify which interactions occur under physiological or oncogenic conditions.

Multiple biophysical, structural, and proteomic studies have refined our understanding by showing that RAS family GTPases exhibit intrinsic, subfamily- and isoform-specific preferences for downstream effectors [31,36–40]. Quantitative binding measurements compiled in Table 1 support these preferences. Because the reported dissociation constant (*K*_D_) values were obtained under diverse experimental conditions—including differences in temperature, ionic strength, nucleotide state, and detection platform—absolute affinities are best interpreted by relative rank order within individual studies. Despite this variability, consistent selectivity trends emerge across effector classes. Canonical RAS subfamily members predominantly recruit RAF kinases, RRAS subfamily proteins (RRAS2 and MRAS) selectively engage PI3Kα, and RAP subfamily proteins (RAP1A and RAP1B) preferentially interact with RalGDS family proteins (Figure 2). These intrinsic preferences are influenced by the fraction of active, GTP-bound protein and the relative binding affinities for each effector. They are further modulated by RAS isoform expression levels, subcellular localization, post-translational modifications, effector availability, and scaffolds or regulatory proteins that facilitate or restrict specific interactions [31,35,41–43]. Together, these factors establish a quantitative hierarchy that determines productive signaling under physiological conditions. This framework explains how RAS family GTPases, despite sharing a conserved molecular switch mechanism, generate distinct signaling outputs and specialized cellular responses, enabling precise regulation of proliferation, survival, differentiation, and migration across diverse cellular contexts.

**Table 1:** Comparative binding affinities of RAS family GTPases for RAF, PI3Kα, and RalGDS

RAS protein	Effector	*K*_D_ (μM)	Strength	Technique	Reference
**RAF1-RBD**					
KRAS	RAF1-RBD	0.018	+++++	FT	[92]
NRAS	RAF1-RBD	0.004	+++++	FT	[92]
HRAS	RAF1-RBD	0.018	+++++	FT	[36]
RRAS	RAF1-RBD	1.1	++	FT	[36]
HRAS	RAF1-RBD	0.16	++++	SF	[93]
HRAS	RAF1-RBD	0.08	+++++	ITC	[94]
RRAS	RAF1-RBD	0.45	++	ITC	[94]
RRAS2	RAF1-RBD	0.63	++	ITC	[94]
RAP1A	RAF1-RBD	0.67	++	ITC	[94]
HRAS	RAF1-RBD	0.066	+++++	SF	[95]
KRAS	RAF1-RBD	0.056	+++++	AS	[96]
KRAS	RAF1-RBD	0.142	++++	FP	[35]
HRAS	RAF1-RBD	0.094	+++++	FP	[35]
NRAS	RAF1-RBD	0.048	+++++	FP	[35]
RRAS	RAF1-RBD	2.29	+	FP	[35]
RRAS2	RAF1-RBD	4.09	+	FP	[35]
KRAS	RAF1-RBD	0.152	+++++	SPR	[47]
KRAS	RAF1-RBD	0.356	++++	SPR	[47]
**PI3Kα (full-length or RBD)**					
RRAS2	PI3Kα (FL)	3.9	+++++	ITC	[39]
MRAS	PI3Kα (FL)	5.3	+++++	ITC	[39]
KRAS	PI3Kα (FL)	16.9	++	ITC	[39]
HRAS	PI3Kα (FL)	20.2	++	ITC	[39]
NRAS	PI3Kα (FL)	22.1	++	ITC	[39]
RRAS	PI3Kα (FL)	27.9	++	ITC	[39]
RRAS2	PI3Kα-RBD	7.8	+++++	ITC	[39]
MRAS	PI3Kα-RBD	5.5	+++++	ITC	[39]
KRAS-G12D	PI3Kα-RBD	12.4	+++	ITC	[39]
KRAS-G12V	PI3Kα-RBD	14.7	+++	ITC	[39]
KRAS	PI3Kα-RBD	23.1	++	ITC	[39]
NRAS	PI3Kα-RBD	24.2	++	ITC	[39]
HRAS	PI3Kα-RBD	29.1	++	ITC	[39]
RRAS	PI3Kα-RBD	36.4	++	ITC	[39]
RRAS	PI3Kα-RBD	11.0	+++	FP	[35]
RRAS2	PI3Kα-RBD	18.1	++++	FP	[35]
KRAS	PI3Kα-RBD	204.7	+	FP	[35]
HRAS	PI3Kα-RBD	84.3	+	FP	[35]
NRAS	PI3Kα-RBD	145.0	+	FP	[35]
**RalGDS-RBD**					
HRAS	RalGDS-RBD	1.0	++	FT	[36]
RAP1A	RalGDS-RBD	0.01	+++++	FT	[36]
RAP1A	RalGDS-RBD	0.08	+++++	ITC	[94]
HRAS	RalGDS-RBD	1.00	++	ITC	[94]
RRAS	RalGDS-RBD	1.59	++	ITC	[94]
RRAS2	RalGDS-RBD	0.45	++++	ITC	[94]
HRAS	RalGDS-RBD	1.0	++	ITC	[95]
MRAS	RalGDS-RBD	3.7	++	ITC	[95]
HRAS	RalGDS-RBD	2.50	++	FP	[35]
KRAS	RalGDS-RBD	1.39	++	FP	[35]
NRAS	RalGDS-RBD	2.84	++	FP	[35]
RRAS	RalGDS-RBD	9.71	+	FP	[35]
RRAS2	RalGDS-RBD	5.78	+	FP	[35]

The table compiles published equilibrium dissociation constants (*K*_D_, μM) for interactions between RAS family GTPases and their effectors. Data are grouped by effector class but retain study-specific measurements, constructs, and methodologies: FT, fluorescence titration; SF, stopped-flow fluorescence; ITC, isothermal titration calorimetry; FP, fluorescence polarization; AS, AlphaScreen; and SPR, surface plasmon resonance. Effector constructs include isolated Ras-binding domains and full-length PI3Kα (p110α/p85 complex). RAS proteins were examined in different nucleotide states (e.g., GTPγS-, GMPPNP-, or mant-nucleotide–loaded forms), which can influence conformational equilibria and effector engagement kinetics and thermodynamics. Reported *K*_D_ values span several orders of magnitude and were obtained under diverse experimental conditions, including differences in buffer composition, ionic strength, pH, temperature, protein concentration, nucleotide analog, and detection platform. Variations in Mg^2+^ concentration, salt levels, reducing agents, labeling strategies, and surface immobilization (for SPR) can affect measured affinity. *K*_D_ values derived from kinetic approaches (SF, SPR), equilibrium thermodynamic measurements (ITC, FT), and competition-based assays (FP, AS) are not strictly interchangeable; therefore, direct comparison of absolute affinities across techniques or laboratories should be interpreted cautiously. Strength was assigned study-wise and subsequently organized within each effector class to reflect relative binding preference rather than absolute cross-study ranking. Within each effector: +++++, dominant/preferred binder(s); ++++, strong; +++, moderate; ++, weak; +, very weak. This framework highlights conserved selectivity trends (classical RAS isoforms for RAF1, RRAS2/MRAS for PI3Kα, and RAP1A/B for RalGDS) while acknowledging methodological variability.

## Switch regions and effector interfaces

**Figure 3:**
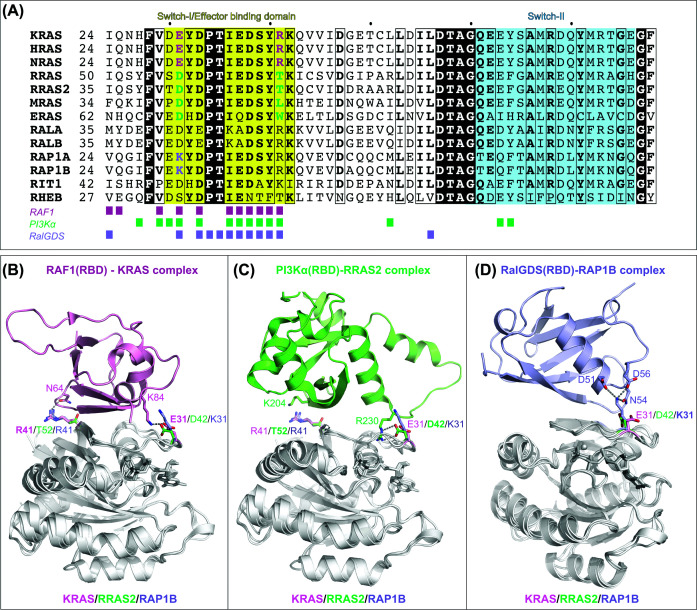
Sequence and structural determinants of RAS subfamily selectivity for downstream effectors (**A**) Sequence alignment of switch regions. Multiple sequence alignment of the switch regions across RAS subfamilies (KRAS, HRAS, NRAS, RRAS, RRAS2, MRAS, ERAS, RALA, RALB, RAP1A, RAP1B, RIT1, and RHEB). The switch-I/effector-binding domain is highlighted in yellow and switch-II in cyan. Key residues contributing to effector selectivity are colored in yellow, green, or purple. Colored squares below the alignment indicate residues that directly interact with RAF1 (pink; PDB: 6VJJ), PI3Kα (green; PDB: 9B4S), or RalGDS (light blue; PDB: 10CG). (B–D) Structural basis of effector selectivity. Crystal structures of RAF1, PI3Kα, and RalGDS RBD domains in complex with their preferred GTPase partners (KRAS, RRAS2, and RAP1B, respectively) are shown (PDB: 6VJJ, 9B4S, and 10CG). For comparison, the other two RAS family members are superposed in light gray, with key side chains highlighted for KRAS (pink), RRAS2 (green), and RAP1B (light blue). (**B**) KRAS–RAF1(RBD) complex: The RAF1 RBD (pink) binds KRAS with high affinity via residues including E31 and R41. E31 forms a critical salt bridge with RAF1 K84, which is absent in RRAS2 (D42; shorter side chain) and repelled in RAP1B (K31; positive charge). KRAS R41 hydrogen bonds with RAF1 N64, a contact lost in RRAS2 due to substitution by T52. (**C**) RRAS2–PI3Kα(RBD) complex: The PI3Kα RBD (green) engages multiple RRAS2 residues, including D42 and T52. D42 forms a salt bridge with PI3Kα R230, and T52 positions favorably near K204. The RRAS2 D42 and T52 residues correspond to E31 and R41 in KRAS and to K31 and R41 in RAP1B. In KRAS, E31’s longer side chain prevents interaction with R230. In RAP1B, K31's positive charge causes repulsion with R230. Bulky R41 in both KRAS and RAP1B, occupying the T52 position in RRAS2, introduces steric and electrostatic clashes, further weakening binding and collectively reducing PI3Kα affinity. (**D**) RAP1B–RalGDS(RBD) complex: The RalGDS RBD (light blue) engages multiple RAP1B residues, including K31, which forms a hydrogen-bond network with D51, N54, and D56, driving high-affinity binding. The corresponding residues in KRAS and RRAS2 are E31 and D42, respectively; in both cases, the acidic side chain generates charge repulsion, preventing the formation of an equivalent hydrogen-bonding network.

Effector engagement by RAS is primarily governed by the switch-I and switch-II regions, two conformationally flexible loops that respond to the nucleotide-bound state [31,44,45]. Switch-I serves as the main effector-binding interface, making the majority of direct contacts, while switch-II provides additional interactions in some cases, together contributing to binding specificity. Upon GTP loading, both switch regions adopt coordinated conformations that expose these critical residues, generating a surface optimized for high-affinity effector interactions. These rearrangements not only present contact points but also define the orientation, kinetics, and selectivity of effector recruitment, enabling isoform- and context-specific signaling. Structural, biochemical, and mutagenesis studies demonstrate that subtle differences in switch-I/II sequences or their conformational dynamics fine-tune effector preferences, providing a mechanistic basis for the distinct engagement patterns among RAS subfamilies (Figure 3A). Together, these nucleotide-responsive switch regions serve as central determinants through which RAS family GTPases translate their activation state into precise and selective cellular signaling outcomes.

## RAF as the primary effector of canonical RAS isoforms

The three RAF kinases, ARAF, BRAF, and CRAF (RAF1), serve as the primary effectors of canonical RAS isoforms KRAS, HRAS, and NRAS [46]. In normal cells, activation of RAF kinases appears to be the principal function of these RAS proteins; other effector interactions, if they occur, appear to be functionally minor under physiological conditions. Biochemical and biophysical measurements summarized in Table 1 demonstrate that canonical RAS isoforms bind the RAF1 RBD with consistently high affinity in the nanomolar range (typically ∼0.004–0.18 μM across FT, ITC, FP, SF, and SPR assays) [35,36,47], establishing the standard benchmark for RAS–effector interactions. In contrast, RRAS, RRAS2, and RAP1A bind RAF1 RBD substantially more weakly, generally in the ∼0.45–4 μM range. This ∼10–100-fold affinity difference, reproducible across independent techniques, quantitatively explains why canonical RAS isoforms are uniquely optimized for RAF recruitment and MAPK activation under physiological conditions. The magnitude and consistency of this difference establish RAF as the principal high-affinity effector of canonical RAS proteins. The high affinity of RAS proteins for RAF kinases enables active RAS to recruit RAF to the plasma membrane, where activation takes place.

Although RAP1 binds the RAF1 RBD with lower affinity than canonical RAS proteins [36], its conserved effector-binding interface made it a valuable surrogate for early structural studies. The first crystal structure of the RAF1 RBD in complex with RAP1 was solved because RAP1 formed better diffraction-quality crystals than RAS [48]. Mutations of two RAS-equivalent residues in RAP1 (E30D and K31E) in the switch-I/effector-binding domain demonstrated the key determinants of high-affinity interaction [49]. The structure revealed that the RBD adopts a β-sandwich ubiquitin-like fold, establishing a structural framework for effector engagement. Subsequent crystallographic and NMR studies with HRAS and KRAS confirmed that the interface and binding mode observed in the RAP1–RAF1 RBD structure accurately reflect authentic RAS–RAF interactions [47,50,51].

The cysteine-rich domain (CRD) of RAF kinases, located next to the RBD, facilitates membrane recruitment and contributes to RAS binding [52,53]. In KRAS–RAF1 (RBD-CRD) complexes, KRAS binds the RBD primarily via switch-I residues, while additional interactions with the CRD further enhance binding affinity by roughly two-fold through conserved contacts in the interswitch region and C-terminal helix [47,54]. Other RAS subfamilies, including RRAS2, MRAS, and RHEB, bind RAF more weakly than even the RAP subfamily [35,36,40,47] (Table 1). Sequence variations and structural divergence in switch-I, switch-II, and the interswitch region disrupt critical contacts required for stable engagement of the RBD and CRD [47], explaining why these noncanonical RAS family GTPases are largely ineffective at activating RAF-dependent MAPK signaling.

In the KRAS–RAF1 RBD complex, multiple interactions collectively support high-affinity binding, including contributions from KRAS switch-I/effector-binding domain residues E31 and R41, where E31 forms a salt bridge with RAF1 K84 and R41 hydrogen bonds with RAF1 N64 (Figure 3B). In contrast, RRAS2 contains D42 and T52 at these positions, preventing equivalent interactions, and RAP1B has K31 in place of E31, introducing charge repulsion at the salt-bridge site. These substitutions account for the markedly lower RBD binding observed in noncanonical RAS isoforms.

The CRD-interacting residues of KRAS are not conserved in other RAS subfamilies, further compounding deficits caused by sequence divergence in the switch-I/II regions. For example, whereas KRAS V45 in the interswitch forms hydrophobic contacts at the KRAS–CRD interface, the equivalent residue in MRAS is a glutamate, which causes a steric clash and disrupts hydrophobic interactions required for CRD recognition [47]. Additional switch-I differences further reduce complementarity for RBD engagement. Similarly, RRAS2 exhibits analogous changes across switch-I, switch-II, and the interswitch loop, collectively weakening both RBD binding and CRD interactions.

Together, these sequence and structural distinctions explain why only canonical RAS isoforms are optimized for robust, dual-domain RAF engagement, whereas MRAS, RRAS2, RAP1, and related noncanonical RAS family members lack the structural and sequence features necessary to efficiently activate RAF-dependent MAPK signaling.

## PI3Kα activation by RRAS2 and MRAS

PI3Kα, composed of the catalytic p110α subunit and the regulatory p85 subunit, is a central signaling node directly downstream of receptor tyrosine kinases [55]. Membrane recruitment of p110α occurs when activated receptor tyrosine kinases bind p85. Binding relieves autoinhibition and also positions the kinase domain at the membrane to catalyze the conversion of PIP_2_ to PIP_3_ [56]. Mice carrying mutations in the PI3Kα RBD that prevent RAS interaction exhibit embryonic defects and impaired RTK-mediated PI3Kα activation during angiogenesis and wound healing, demonstrating that RAS family proteins contribute to PI3Kα signaling under specific physiological conditions [57], though which RAS family proteins are involved remains unclear.

In contrast with RAF activation, RAS proteins may play a relatively minor role in PI3Kα activation, as membrane recruitment and activation by relieving autoinhibition are mediated by activated RTKs. RAS proteins may stabilize PI3Kα complexes in the membrane and so extend the duration of signaling or perhaps localize PI3Kα to regions of the membrane that are rich in PIP_2_. Accumulation of PIP_3_ subsequently recruits PH-domain–containing proteins such as AKT and PDK1, initiating the PI3K–AKT–mTOR signaling cascade that promotes cell survival and growth. Consistent with a modulatory role for RAS proteins, RASless mouse embryonic fibroblasts lacking KRAS, HRAS, and NRAS retain basal and growth factor–induced AKT phosphorylation, particularly upon PTEN loss, indicating that PI3K signaling can occur independently of canonical RAS isoforms [58].

Quantitative binding data compiled in Table 1 reveal a distinct hierarchy for PI3Kα engagement. RRAS2 and MRAS bind full-length PI3Kα with *K*_D_ values of approximately 3.9–5.3 μM, whereas canonical KRAS, HRAS, and NRAS bind more weakly (∼16.9–22.1 μM) [38,39]. A similar trend is observed with the isolated PI3Kα RBD. Thus, RRAS2 and MRAS display roughly a four- to six-fold affinity advantage over canonical RAS isoforms. This difference, although smaller than that observed for RAF, is sufficient to bias physiological PI3Kα activation toward RRAS2/MRAS at basal RAS-GTP levels. RRAS binds PI3Kα weakly, similar to canonical RAS [39]. This difference likely stems from a switch-I substitution, with T41 in RRAS2 and S56 in RRAS, where the extra methyl group in threonine enhances interaction with PI3Kα, and additional sequence differences near the interface may further influence binding strength. ERAS, expressed exclusively in embryonic cells, has been shown to interact with PI3Kα, whereas most other RAS subfamily members show little or no detectable binding to PI3Kα [38,59].

Because of their low binding affinity, canonical RAS proteins are unlikely to activate PI3Kα in normal cells. So far, there is no direct evidence that KRAS, HRAS, or NRAS activate PI3Kα during normal signaling. However, there is now clear evidence that oncogenic RAS mutants can engage PI3Kα in cancer cells, possibly due to abnormally elevated RAS-GTP levels associated with these mutations, as well as increased affinity for PI3Kα for specific oncogenic mutants such as KRAS-G12D and KRAS-G12V [39,60,61]. These findings were facilitated by the development of the small-molecule drug BBO-10203, which prevents canonical RAS proteins from binding to PI3Kα and inhibits PI3Kα signaling in tumor cells expressing KRAS mutants [60]. These observations indicate that the interaction between canonical RAS proteins and PI3Kα is a neomorphic function of oncogenic mutants. As discussed below, this may also apply to RalGDS and other putative RAS effectors.

The higher affinity of RRAS2 and MRAS is determined by a small number of unique sequence features within the switch regions that optimize contacts with the p110α RBD. In the RRAS2–PI3Kα complex, key interactions include RRAS2 D42 forming a stabilizing salt bridge with p110α R230, while RRAS2 T52 allows favorable packing near p110α K204 [39] (Figure 3C). In canonical KRAS, the equivalent residue to D42 is E31, whose longer side chain cannot form a salt bridge with R230, and in RAP1B, K31 introduces charge repulsion with the same residue. Similarly, the T52-equivalent residue in KRAS and RAP1B is R41, whose longer side chain generates steric and electrostatic clashes with p110α K204. Consistent with this structural logic, mutating non-conserved KRAS residues toward their RRAS2 counterparts modestly enhances binding, with the single E31D substitution nearly restoring KRAS affinity for PI3Kα to levels comparable to RRAS2/MRAS [39].

Collectively, these findings indicate that RRAS2 and MRAS are well-positioned to function as high-affinity activators of PI3Kα, efficiently coupling their GTP-bound states to PI3K–AKT signaling to amplify RTK signaling. In contrast, canonical RAS isoforms engage PI3Kα inefficiently at physiological RAS-GTP levels, reinforcing a hierarchy in which intrinsic binding affinities encode effector selectivity. Together with RAF signaling, this paradigm illustrates how closely related RAS-family GTPases are structurally tuned to drive distinct downstream signaling pathways.

## RalGDS activation by RAP1 isoforms

RalGDS (Ral guanine nucleotide dissociation stimulator) is a GEF that activates the RAL subfamily GTPases RALA and RALB by catalyzing GDP–GTP exchange [62,63]. Acting downstream of RAS family GTPases, RalGDS transduces upstream signals to drive RAL-dependent processes [64–66], including vesicle trafficking, cytoskeletal remodeling, cellular adhesion, and migration. Dysregulation of this pathway contributes to tumor progression and metastatic behavior. The RalGDS RA (Ras-Association) domain is a ∼80–100-residue module that binds RAS-family GTPases, primarily recognizing the switch-I region, and is structurally analogous to classical RBDs. Although RAS-binding domains were historically classified as RBDs or RA domains based on sequence similarity and functional context, structural studies have shown that both share a ubiquitin fold and engage RAS using a conserved binding mode, supporting their classification within a unified RBD family [31].

RAP1 was initially identified as a preferred binding partner of RalGDS and was subsequently shown to function as an efficient upstream activator of RAL signaling [36,67]. Quantitative measurements summarized in Table 1 support this model, showing that RAP1A binds the RalGDS RBD with submicromolar affinity (∼0.1–0.5 μM), whereas canonical RAS isoforms bind weakly, with *K*_D_ values beginning around ∼1 μM and extending into the low micromolar range. Consistent with these observations, isothermal titration calorimetry confirms RalGDS strong selectivity for RAP1 isoforms: RAP1A and RAP1B bind with high affinity (*K*_D_ ≈ 0.3–0.5 μM), whereas KRAS, HRAS, and NRAS bind weakly (*K*_D_ ≈ 6–12 μM), making them inefficient activators at physiological RAS-GTP levels (unpublished data). Together, these measurements define an approximately order-of-magnitude affinity advantage for RAP1 over canonical RAS proteins. As observed for the reciprocal RAS–RAF interaction, this quantitative difference is sufficient to bias effector engagement under normal conditions, supporting a model in which RAP1 isoforms serve as the principal high-affinity activators of RalGDS signaling.

Structural studies provide a mechanistic basis for this isoform selectivity [68,69]. The RalGDS RBD engages RAP1B via a switch-I–centered interface, with K31 playing a central role by forming a hydrogen-bonding network with D51, N54, and D56 of RalGDS that stabilizes high-affinity binding (Figure 3D). In canonical RAS and RRAS subfamilies, substitution of this RalGDS K31 with acidic residues (E31 in RAS or D42 in RRAS2) introduces charge repulsion and disrupts the hydrogen-bond network, weakening the interaction. Binding studies show that RAS binds the RAF1 RBD and RAP1A binds the RalGDS RBD with ∼100-fold higher affinity than the reciprocal interactions, underscoring residue 31 as a principal determinant of selectivity between RAF and RalGDS [70]. Swapping this residue shifts effector preference: K31E in RAP1A increases RAF binding ∼15-fold and decreases RalGDS binding ∼20-fold, whereas E31K in RAS reduces RAF binding ∼5-fold and enhances RalGDS binding ∼5-fold. Crystallization of HRAS in complex with the RalGDS RBD required the E31K mutation, which restores electrostatic and hydrogen-bond complementarity at the interface and recapitulates the binding mode observed with RAP1. These findings underscore how subtle differences in switch-I residues dictate effector preference. Together, the RAP1–RalGDS pathway represents a distinct high-affinity, isoform-specific signaling module within the RAS family. By coupling RAP1 activation to RAL GTPases largely independently of canonical RAS effectors, this axis enforces a functional division of labor among closely related GTPases.

## Oncogenic RAS-GTP levels and bypass of effector selectivity

Physiological RAS signaling is regulated by isoform-specific effector affinities that constrain pathway output under normal conditions. Quantitative modeling and proteomic analyses indicate that, at basal RAS-GTP levels, signaling primarily flows through high-affinity effectors [35,37,71], thereby establishing a defined hierarchy of pathway engagement for each RAS-family GTPase. Oncogenic mutations at codons G12, G13, or Q61 impair intrinsic and GAP-mediated GTP hydrolysis, locking RAS in a GTP-bound state and substantially increasing the cellular pool of active RAS. Furthermore, in many cancers, mutant alleles are also amplified or overexpressed, further elevating total RAS abundance. This supraphysiological increase in both the abundance and the accumulation of active RAS-GTP can saturate primary high-affinity binding sites and permit engagement of secondary, lower-affinity effectors that are rarely engaged by wild-type RAS, thereby contributing to carcinogenesis.

This expansion of effector engagement effectively overrides the intrinsic isoform-specific preferences encoded within individual RAS proteins. Canonical RAS proteins, particularly oncogenic KRAS mutants, can thus engage pathways that are weakly coupled under physiological conditions; for example, oncogenic KRAS shows enhanced coupling to PI3Kα. Structural and quantitative binding studies further reveal that KRAS-G12D and KRAS-G12V not only maintain a higher fraction of GTP-bound protein but also bind the PI3Kα RBD with approximately two-fold higher affinity than wild-type KRAS (*K*_D_ ≈ 12–15 μM versus ∼23 μM; Table 1) [39], thereby directly contributing to increased PI3Kα activation in the oncogenic context. Although this increase does not reach the affinity levels of RRAS2/MRAS, it narrows the gap sufficiently to promote more efficient PI3Kα engagement when combined with elevated RAS-GTP abundance. Of note, these two oncogenic variants are the most common in human cancer, suggesting that their relatively high affinity for PI3Kα may confer a selective advantage through hypermorphic and potentially neomorphic signaling effects.

The resulting expansion of effector engagement rewires downstream signaling networks. Oncogenic KRAS can simultaneously activate the MAPK, PI3K–AKT, and RALA/B pathways, thereby bypassing the normal hierarchy of isoform-restricted effector selectivity. This promiscuous signaling amplifies downstream outputs and drives the enhanced proliferation, survival, cytoskeletal remodeling, and migratory behaviors characteristic of transformed cells. Importantly, these observations underscore that effector selectivity is not absolute but is constrained by the abundance of active RAS-GTP under physiological conditions.

Mass spectrometry analysis of proteins that bind members of the RAS family in their active state revealed a specific interaction among MRAS, SHOC2, and PP1C [72]. The ternary complex formed by these three proteins dephosphorylates negative phosphorylation sites on RAF proteins bound to RAS at the plasma membrane, enabling efficient RAF dimerization and activation [73,74]. Subsequent structural and biochemical analysis revealed that MRAS binds SHOC2 and PP1C in its GTP-bound state and utilizes sequences in the switch and interswitch regions, as well as residues at the N- and C-termini [75–78]. Furthermore, MRAS binds to SHOC2/PP1C with significantly higher affinity than other RAS proteins [75–78], as suggested by the original mass spectrometry analysis. However, the biological relevance of MRAS remains unclear. Mice lacking MRAS are viable, but mice lacking SHOC2 do not survive during development [79,80]. This suggests that other RAS proteins can substitute for MRAS, despite their lower affinity. Likewise, many cancer cells depend strongly on SHOC2, but very few, if any, depend on MRAS, again suggesting that other RAS proteins can substitute. Alternatively, SHOC2 may have additional essential functions that are independent of RAS signaling. Of note, Q61 mutants of RAS proteins bind SHOC2 with relatively high affinity, and cancer cells expressing these mutants are highly sensitive to SHOC2 inhibition [45,77]. These observations suggest that oncogenic RAS mutants may substitute for the specialized role of MRAS in SHOC2 complex formation, representing another instance in which elevated RAS-GTP levels expand effector utilization, but in this case, the oncogenic variant is a different member of the RAS family.

Collectively, these findings support a model in which each RAS-family GTPase exhibits a defined effector-binding profile at basal RAS-GTP levels, shaped not only by intrinsic affinity but also by RAS and effector abundance and by nucleotide turnover rates. Oncogenic mutations elevate RAS-GTP beyond normal thresholds, broadening the effector repertoire and enabling canonical RAS isoforms to co-opt signaling pathways typically engaged by other high-affinity RAS family members, as suggested by Rodriguez-Viciana and colleagues [81], who first identified the spectrum of RAS family potential effectors. This bypass of intrinsic effector hierarchy provides a mechanistic explanation for the pervasive oncogenic potency of KRAS mutations and their capacity to drive tumorigenesis and therapeutic resistance.

## Subcellular localization and tissue-specific expression in isoform-specific signaling

Beyond intrinsic binding affinities, subcellular localization and tissue-specific expression of RAS-family GTPases and their effectors jointly shape signaling outputs by controlling where, when, and at what concentration each isoform encounters its effectors. In this framework, intrinsic effector preferences establish a baseline hierarchy of interactions that is further refined by spatial and quantitative constraints, reinforcing some interactions while limiting others in a context-dependent manner.

Canonical RAS isoforms are primarily targeted to the plasma membrane through C-terminal lipid modifications, whereas noncanonical members access additional compartments, including the Golgi, endomembranes, lysosomes, mitochondria, and cytoskeletal structures [82,83]. Effector proteins likewise exhibit distinct localization patterns, which can bias interactions toward specific RAS isoforms. Although direct quantitative evidence linking localization to effector selectivity remains limited, spatial segregation likely reinforces the intrinsic preferences established by switch region interfaces and binding affinities. Several examples illustrate this principle. SIN1, an effector protein that is part of the mTORC2 complex, interacts preferentially with the KRAS4A splice variant through an extended RBD/PH-domain interface, potentially facilitated by compartmental colocalization [84]. KRAS4A can also access the outer mitochondrial membrane through its palmitoylation cycle and has been proposed to interact with hexokinase 1, linking RAS signaling to metabolism [85]. RIT1 can localize to the plasma membrane despite lacking a CaaX motif but can also bind to the spindle assembly checkpoint in the cytoplasm. Its ability to shuttle between these two compartments appears to be regulated by phosphorylation [86]. KRAS proteins themselves are thought to remodel lipids within the plasma membrane, creating PIP_2_ clusters [87] that may interact with the CRD of RAF proteins and affect their activation and also serve as a substrate for PI3Kα following RAS recruitment.

At the tissue level, differential isoform expression shapes which parts of the affinity hierarchy are most consequential in a given cell type. KRAS typically accounts for 50%–75% of total RAS protein across most tissues, whereas HRAS and NRAS are more restricted [41]. Noncanonical members, including RRAS2, MRAS, and RAP1 isoforms, also vary across cell types and contribute to context-dependent signaling [37,88,89]. Because more abundant isoforms contribute proportionally to the active RAS-GTP pool, tissue expression patterns bias which interactions within the baseline hierarchy are most effectively realized in a given cellular context [37,41]. Cancer mutation patterns broadly reflect this landscape: KRAS predominates in pancreatic, colorectal, and lung cancers; NRAS in melanoma and hematopoietic malignancies; and HRAS in bladder and head-and-neck cancers, consistent with their relative tissue-specific expression [90]. The pancreas provides a notable exception. Although all three canonical isoforms are expressed at comparable levels, mutations are overwhelmingly KRAS-driven [41,91], indicating that factors beyond abundance, such as isoform-specific signaling compatibility and tissue-specific selective pressures, also shape oncogenic fitness.

Effector expression further modulates this landscape. RAF, PI3K, and RalGDS family proteins are differentially expressed across tissues, creating a competitive environment in which both affinity and protein concentration influence signaling output [31,33,37]. Systems-level analyses suggest that effectors are generally present in excess relative to active RAS-GTP, so variation in isoform and effector levels can bias pathway utilization [37]. As a result, lower-affinity interactions may still contribute when either partner is locally abundant, whereas higher-affinity interactions tend to dominate when protein levels are limiting [42]. Together, these observations indicate that intrinsic effector preferences establish a baseline hierarchy that is refined by both subcellular localization and tissue-specific expression, creating a context-dependent framework for isoform-specific RAS signaling and helping explain how distinct signaling outputs emerge across cell types and disease states.

## A revised model of RAS family signaling

The biochemical, structural, and cellular evidence reviewed here supports a model in which the RAS family is organized around a clear division of signaling labor. Under physiological conditions, each subfamily is structurally tuned for a preferred effector: canonical RAS for RAF, RRAS2 and MRAS for PI3Kα, and RAP1 for RalGDS (Figure 2). This specialization is encoded in the switch regions, reinforced by subcellular localization, and further shaped by tissue-specific expression. Together, these features establish a hierarchy of effector preferences that constrains signaling outputs and ensures that each RAS subfamily contributes to distinct downstream signaling outputs.

A key implication of this framework is that several effector interactions historically attributed to canonical RAS, including PI3Kα and RalGDS activation, are more accurately explained as functions of other RAS subfamilies under physiological conditions and as neomorphic or hypermorphic properties of oncogenic RAS in disease. The RAS–PI3Kα interaction illustrates this clearly: weak in vitro and largely dispensable under basal conditions, yet essential for oncogenic RAS-driven tumorigenesis and selectively targetable in KRAS-mutant cancers [57,60,61]. This shift arises through two complementary mechanisms. First, impaired GTP hydrolysis elevates RAS-GTP above physiological levels, enabling progressive engagement of lower-affinity effectors in order of affinity. Second, specific variants directly alter effector interactions; for example, KRAS-G12D and KRAS-G12V bind the PI3Kα RBD with approximately two-fold higher affinity than wild-type KRAS [39], narrowing the gap with RRAS2 and MRAS and suggesting that enhanced PI3Kα coupling contributes to their selective advantage. Together, these effects indicate that interactions that are minor under physiological conditions can become functionally significant in oncogenic settings, a principle that likely extends to other low-affinity RAS–effector interactions.

In vitro binding affinities provide a useful quantitative baseline for RAS–effector interactions but must be interpreted in the context of cellular physiology, where additional regulatory layers, including membrane anchoring, protein abundance, multivalent interactions, post-translational modifications, and feedback regulation, influence signaling outcomes. Membrane confinement increases effective local concentrations and promotes repeated encounters between RAS and its effectors, while auxiliary domains and regulatory inputs can stabilize interactions beyond what solution-phase affinities alone would predict. These factors are likely to preserve the intrinsic rank order of effector preferences but can narrow the differences between them, allowing lower-affinity interactions to become functionally relevant under specific conditions. As a result, interactions that appear weak in isolation can contribute to signaling when RAS-GTP levels are elevated or when cellular context stabilizes otherwise transient complexes. Taken together, these observations indicate that in vitro affinities and cellular outcomes are complementary: biochemical measurements define baseline interaction potential, whereas cellular context determines how and under what conditions those interactions are realized in signaling.

Recognizing effector selectivity as a quantitative, context-dependent property of RAS signaling refines our understanding of RAS biology and highlights new therapeutic opportunities. In particular, the ability of oncogenic RAS proteins to engage effectors that wild-type RAS rarely interacts with provides therapeutic opportunities that have not been previously exploited.

## Open Access

Open access for this article was enabled through an open access agreement between the Biochemical Society/Portland Press and the University of California.

## Abbreviations

CRD: cysteine-rich domain
GAPs: GTPase-activating proteins
GEFs: guanine nucleotide exchange factors
RalGDS: Ral guanine nucleotide dissociation stimulator
